# Complete Genome Sequence of *Pseudoalteromonas* sp. Strain LC2018020214, a Bacterium Isolated from Natural Seawater

**DOI:** 10.1128/MRA.00075-21

**Published:** 2021-03-18

**Authors:** Pish Wattanadilokchatkun, Pattanapon Kayansamruaj, Jiao Pan

**Affiliations:** a Institute of Evolution and Marine Biodiversity, KLMME, Ocean University of China, Qingdao, Shandong Province, China; b Faculty of Fisheries, Kasetsart University, Bangkok, Thailand; University of Southern California

## Abstract

*Pseudoalteromonas* is a genus widely distributed in the ocean and displays antibacterial and antifouling activities. We isolated a *Pseudoalteromonas* sp. strain (LC2018020214) from coastal water of Qingdao, China, and assembled its complete genome. The genome consists of two circular chromosomes with lengths of 3,700,777 bp and 817,517 bp, respectively, and 3,866 coding sequences.

## ANNOUNCEMENT

*Pseudoalteromonas* is a group of Gram-negative marine bacteria that can synthesize biologically active molecules (1–3). Some metabolites of *Pseudoalteromonas* have antibacterial and antifouling activities, and the bacteria can also prey on other bacteria and affect the formation of biofilm from some marine organisms (4–9). *Pseudoalteromonas* sp. strain LC2018020214 was isolated from the natural seawater of Qingdao in Shandong Province, China (36.06°N, 120.37°E; 3.4°C; 2 February 2018). This strain could be used for culturing various bacterivorous ciliated protozoa.

To isolate the bacterium, a 10-cm^2^ piece of sea lettuce was collected and immersed in 30 ml seawater on site. Then, 100 μl of seawater was spread onto a marine LB agar plate (catalog [cat.] no. 8290; Solarbio, China) and cultured overnight in a 25°C incubator. A single colony was streaked onto a new marine LB agar plate, inoculated into 6 ml marine LB broth in an 18 × 150-mm test tube for 14 h at 25°C with shaking at 200 rpm. Genomic DNA was then extracted using the MasterPure Complete DNA and RNA purification kit (cat. no. MC85200; Lucigen, USA) and purified using genomic DNA Clean & Concentrator-10 (cat. no. D4010; Zymo Research, USA). Genomic DNA was quantified using a Qubit v3.0 fluorometer and quality checked on a Nano-300 microspectrophotometer. The identification of *Pseudoalteromonas* sp. LC2018020214 was done with a GEN III MicroPlate (cat. no. 1030; Biolog, USA) and 16S rRNA gene sequencing (PCR primers 5′-AGAGTTTGATCCTGGCTCAG-3′ and 5′-CGGTTACCTTGTTACGACTT-3′; https://www.ezbiocloud.net/taxonomy), and the strain was stored in LB medium with 10% glycerol at −80°C.

For Illumina sequencing, the genomic DNA library was prepared with a TruSeq Nano kit (cat. no. 20015964; Illumina, USA) and sequenced using paired-end 150-bp reads with a NovaSeq 6000 sequencer at Berry Genomics, Inc. (Beijing, China). Clean reads (66.18 million) were produced after a quality check, and reads/adaptors were trimmed with FastQC v0.11.8 and Trimmomatic v0.32 (10, 11). For Nanopore long-read sequencing, a library was prepared with the ligation sequencing kit (SQK-LSK109; Oxford Nanopore Technologies) and was loaded into an R9.4.1 flow cell (Oxford Nanopore) on a PromethION platform at NextOmics Biosciences (Wuhan, China). We used Guppy v3.3.3 for base calling, filtered out reads with a mean base quality score of <8 and length of <1,000 bp using NanoFilt v2.7.1, and obtained 320,750 high-quality reads with *N*_50_ subread length of 21,317 bp (12). All software used the default parameters unless otherwise specified.

We used Unicycler v0.4.8 (13) to assemble the genome sequence of *Pseudoalteromonas* sp. LC2018020214 using the high-quality short and long reads with the default settings. The genome was annotated using the NCBI Prokaryotic Genome Annotation Pipeline (14). The complete genome contains two circular chromosomes (3,700,777 and 817,517 bp) with GC content of 39.35% and 38.80%, respectively, mean long-read sequence depth of 597×, and no plasmid sequences. BUSCO v4.1.4 (15) was used to assess the genome quality with the database of bacteria_odb10, and the coverage rate of complete universal single-copy orthologs in the genome was 98.4%. In total, we predicted 3,866 coding sequences and 135 RNA genes (28 rRNAs, 103 tRNAs, and 4 noncoding RNAs [ncRNAs]).

### Data availability.

This whole-genome sequence has been deposited in NCBI with BioProject and BioSample accession no. PRJNA687783 and SAMN17158607, respectively, and GenBank accession no. CP066804.1 and CP066805.1. The SRA accession numbers of the Illumina and Nanopore reads are SRR13307307 and SRR13307390, respectively.
